# Whole-blood ribonucleic acid sequencing analysis in methemoglobinemia: a case report

**DOI:** 10.1186/s13256-023-03976-0

**Published:** 2023-06-10

**Authors:** Chikai Mitsuhara, Yuki Togami, Tomoya Hirose, Shunichiro Nakao, Hiroshi Ito, Hisatake Matsumoto, Hiroshi Ogura, Daisuke Okuzaki, Jun Oda

**Affiliations:** 1 Department of Traumatology and Acute Critical Medicine, Osaka University Graduate School of Medicine, 2-15 Yamadaoka, Suita City, Osaka 565-0871 Japan; 2 Laboratory of Human Immunology (Single Cell Genomics), WPI Immunology Research Center, Osaka University, Osaka, Japan; 3 Genome Information Research Center, Research Institute for Microbial Diseases, Osaka University, Osaka, Japan; 4 Department of Acute Medicine and Critical Care Medical Center, National Hospital Organization Osaka National Hospital, Osaka, Japan

**Keywords:** Case report, Methemoglobin, RNA sequencing, Hydrogen peroxide

## Abstract

**Introduction:**

Methemoglobinemia is a condition in which methemoglobin is increased and the oxygen carrying capacity of tissues is decreased, causing a lack of oxygen to the whole body. RNA (ribonucleic acid) sequencing technologies have made it possible to systematically examine how the human transcriptome responds to invasive pathologies. To our knowledge, no previous studies have reported the results of RNA sequencing in a patient with methemoglobinemia. We describe the analysis of RNAs from the whole blood of a patient with methemoglobinemia.

**Case presentation:**

A 31-year-old Japanese man was brought to our hospital with symptoms of dyspnea due to inhalation of gas from an acetic acid phosphonitrate storage tank at a factory. The nitrogen oxide concentration measured around the storage tank was over 2500 ppm, and he witnessed orange–brown smoke at that time. After entering the area and taking a few breaths, he suddenly became unwell, with dyspnea and numbness in his extremities. He was evacuated from the area within a few minutes, at which time he was suffering from whole-body cyanosis and was still aware of the above symptoms. On arrival at the hospital, his respiration rate was 18 breaths/minute, and his SpO_2_ ranged from 80% to 85% on 15 L/minute of oxygen by mask (2.5 hours postexposure). Arterial blood gas testing revealed a methemoglobin level of 23.1%. After the administration of methylene blue, the patient’s methemoglobin level normalized and his symptoms improved. Chest X-ray and chest computed tomography showed no evidence of pulmonary edema or interstitial pneumonia, and no other abnormal findings were observed. RNA sequencing was performed on the blood samples obtained at the time of the visit, with the blood sample collected on day 5 used as a control. To our knowledge, the present study is the first to describe the analysis of RNAs from the whole blood of a patient with methemoglobinemia. The RNA sequencing analysis showed that an activated “hydrogen peroxide catabolic process” may be associated with the pathogenesis of methemoglobinemia.

**Conclusion:**

The results reported in the present study may explain the pathogenesis of methemoglobinemia.

**Supplementary Information:**

The online version contains supplementary material available at 10.1186/s13256-023-03976-0.

## Introduction

Acute poisoning by inhalation of nitrogen oxides, such as nitrogen dioxide, is known to be fatal, with a high probability of causing severe pulmonary edema and methemoglobinemia [1]. Methemoglobinemia is a disorder of the red blood cells. Methemoglobin is formed when the iron atom in hemoglobin loses one electron, changing from Fe^2+^ or the reduced state to Fe^3+^ or the oxidized state. Methemoglobin is always present in low concentrations in the body, but methemoglobinemia is defined as an abnormally elevated methemoglobin concentration of 1% or more. Since the late 1990s, technologies and knowledge bases have been developed, including microarrays [2] and sequencing [3]. These technologies have made it possible to systematically examine how the human transcriptome (for example, messenger RNAs) responds to invasive pathologies [4]. To our knowledge, no previous studies have reported the results of RNA sequencing (RNA-seq) in a patient with methemoglobinemia. Therefore, we herein describe for the first time, the analysis of RNA from the whole blood of a patient with methemoglobinemia.

## Case presentation

The patient was a 31-year-old Japanese man with no specific past medical history or family history. He worked as an inspection worker at a metal fabrication plant. He had smoked about ten cigarettes per day for 10 years and was an occasional drinker. Orange smoke rose from a storage tank containing “phosphoric nitric acetic acid” at his metal fabrication plant. Dressed in protective clothing and a protective mask, the patient entered the smoke-filled area to control the smoke being produced. The nitrogen oxide concentration measured around the storage tank was over 2500 ppm, and he witnessed orange–brown smoke at that time. After entering the area and taking a few breaths, he suddenly became unwell, with dyspnea and numbness in his extremities. He was evacuated from the area within a few minutes, at which time he was suffering from whole-body cyanosis and was still aware of the above symptoms. His colleague called for emergency services, and he was transported to an emergency hospital. On arrival at that hospital, his respiration rate was 18 breaths/minute, and his oxygen saturation (SpO_2_) ranged from 80% to 85% on 15 L/minute of oxygen by mask (2.5 hours postexposure). Arterial blood gas testing at the same time showed a high methemoglobin level of 23.1%. Accordingly, he was diagnosed as having methemoglobinemia. The hospital did not have any methylene blue in stock; thus, 3 hours after the initial emergency call, he was transferred to our center, which had methylene blue on hand.

At 4.5 hours after exposure, when the patient was transported to our center, his whole-body cyanosis had improved and his subjective symptoms had disappeared. His consciousness was clear and his blood pressure was stable. However, his SpO_2_ remained low at 90% on 15 L/minute of oxygen by mask, and his respiratory rate was still 18 breaths/minute. Arterial blood gas testing showed that his methemoglobin level was still high at 12.5%. We diagnosed him as having toxic methemoglobinemia due to nitrogen oxide inhalation because there were no other factors that would result in methemoglobinemia. The patient had been on high-flow oxygen since the previous hospital, and although his methemoglobin level had decreased, it was still symptomatic. Consequently, we elected to initiate treatment for methemoglobinemia by administering methylene blue according to package inserts. Methylene blue (total of 100 mg) was administered intravenously. Ten minutes after the initial intravenous administration of methylene blue (50 mg), his methemoglobin level had improved to 6.0%, and 10 minutes after an additional intravenous administration of methylene blue (50 mg), his methemoglobin level had almost normalized to 1.8%. The administration of methylene blue caused his SpO_2_ to improve remarkably; just after its administration, his SpO_2_ was 100% on 5 L/minute of oxygen. Chest X-ray and chest computed tomography (CT) showed no evidence of pulmonary edema or interstitial pneumonia, and no other abnormal findings were observed. He was admitted to our center for follow-up.

After admission, blood tests were conducted frequently, but no re-elevation of his methemoglobin level was observed, and other parameters remained consistently normal. Furthermore, his subjective symptoms did not reappear, and his respiratory status, including his respiratory rate and SpO_2_, was consistently stable without the administration of oxygen. A daily chest X-ray showed no new appearance of ground-glass opacities or consolidation. Whole-blood RNA samples were collected on the first (4.5 hours after exposure), third, and fifth days of admission to our hospital. His general condition remained stable, and he was discharged on day 5 after admission. The diagnosis of methemoglobinemia was straightforward, and there were no adverse events associated with the administration of the methylene blue.

We followed him as an outpatient for 3 months after discharge. At each monthly visit, we conducted chest CT scans and a respiratory function test. Chest CT showed no new ground-glass opacities or consolidation, and respiratory function tests showed no obstructive or restrictive ventilatory impairment. At the 3 month outpatient visit, the patient showed no problems resulting from either subjective symptoms or objective findings, so follow-up was terminated. No adverse effects of methemoglobinemia or complications from the treatment were observed. The timeline of this case is presented in Table 1.

**Table 1:** The timeline of this case

Time, day	Event
X	The patient took in a few breaths of orange–brown smoke
X + a few minutes	The patient became unwell, with dyspnea and numbness in his extremities
X + 2 hours 30 minutes	The patient was suffering from whole-body cyanosis SpO_2_: 80–85% (15 L/minutes of oxygen), respiratory rate: 18/minute MetHb level in arterial blood gas testing: 23.1%
X + 4 hours 30 minutes	The patient was transported to our center His whole-body cyanosis had improved, and his subjective symptoms had disappeared SpO_2_: 90% (15 L/minutes of oxygen), respiratory rate: 18/minute MetHb level in arterial blood gas testing: 12.5% Whole-blood RNA samples were collected
X + 4 hours 40 minutes	Methylene blue (100 mg) was administered intravenously
X + 4 hours 50 minutes	MetHb level in arterial blood gas testing: 6.0%
X + 5 hours	MetHb level in arterial blood gas testing: 1.8% SpO_2_: 100% (5 L/minutes of oxygen) Chest X-ray and chest CT showed no abnormal findings
X + 2 days	Whole-blood RNA samples were collected
X + 4 days	Whole-blood RNA samples were collected He was discharged
X + 1 month	Chest CT and respiratory function tests showed no abnormal findings
X + 2 months	Chest CT and respiratory function tests showed no abnormal findings
X + 3 months	Chest CT and respiratory function tests showed no abnormal findings

*MetHb* methemoglobin, *CT* computed tomography

### RNA sequencing results

**Fig. 1:**
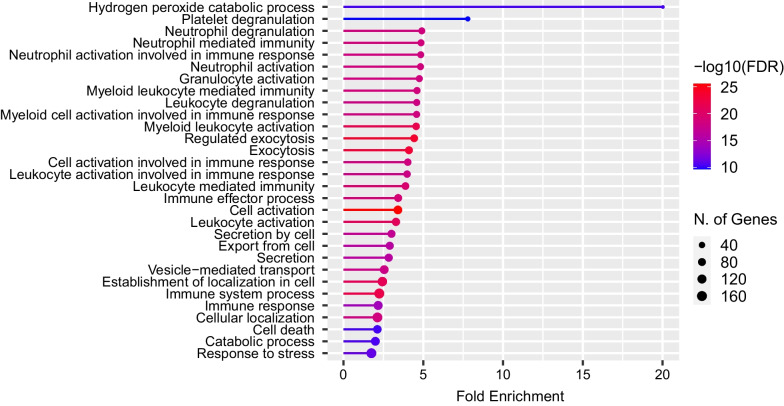
A lollipop plot of the top 30 most enriched Gene Ontology terms that were significantly upregulated in this patient. The length of the bar indicates fold enrichment, the color indicates −log10 (FDR), and the size of the circle at the tip indicates the number of genes involved. *FDR* false discovery rate

RNA-seq identified 26,255 RNAs, 19,000 of which were protein-coding RNAs. As the patient’s symptoms had improved by day 5, the sample collected on day 5 was used as a control and compared with the sample obtained at the time of admission. Among the 2000 RNAs with the highest mean absolute deviation, 473 had a fold change of ≥ 1.5. In the Gene Ontology (GO) enrichment analysis, as shown in Fig. 1, the highest fold enrichment was observed for the hydrogen peroxide catabolic process, followed by platelet degranulation, neutrophil degranulation, neutrophil-mediated immunity, neutrophil activation involved in immune response, and neutrophil activation. A GO analysis using RNA-seq data showed activation of the hydrogen peroxide catabolic process (details are shown in the Additional file 1: Methods).

## Discussion

We experienced a case of methemoglobinemia without pulmonary edema due to inhalation of high concentrations of nitrogen oxides. In general, nitrogen dioxide is colorless at low concentrations and orange–brown at high concentrations. In this case, the nitrogen oxide concentration measurement around the storage tank was over 2500 ppm, and the patient witnessed orange–brown smoke, indicating that the patient may have inhaled high concentrations of nitrogen dioxide. Inhalation of nitrogen dioxide at 150–300 ppm is associated with severe pulmonary edema [5]. It has been reported in a rat model that the development of pulmonary edema depends on the duration and concentration of exposure to nitrogen dioxide, whereas nitrogen dioxide itself does not contribute to the development of methemoglobinemia [6]. In the present case, however, although the patient was assumed to have inhaled nitrogen dioxide at a sufficient concentration to cause pulmonary edema, the patient developed methemoglobinemia rather than pulmonary edema.

Methemoglobinemia occurs as a result of the transfer of a single electron from the iron atom present in hemoglobin to an oxidant, resulting in the conversion of iron from its reduced or ferrous (Fe^2+^) state to its oxidized or ferric (Fe^3+^) state [1]. By acting as an electron donor, methylene blue facilitates the reduction of methemoglobin [7]. The patient was initially transported to another hospital before being brought to our hospital, and unfortunately, it took some time before the methylene blue was administered. In such cases, methylene blue should be administered as soon as possible.

A GO enrichment analysis has shown the presence of the “hydrogen peroxide catabolic process,” which is related to chemical reactions and pathways resulting in the breakdown of hydrogen peroxide in patients with methemoglobinemia [8]. This suggests that hydrogen peroxide may be involved in the reaction during the recovery phase when methemoglobin is reduced to hemoglobin [7]. As an industrial method of controlling nitrogen oxide, a scrubbing solution containing hydrogen peroxide water is used, as shown in the following reaction equation [9].

$${\text{3NO}}_{{2}} + {\text{ H}}_{{2}} {\text{O}} \Leftrightarrow {\text{2HNO}}_{{3}} + {\text{ NO}}$$

$${\text{2NO }} + {\text{ HNO}}_{{3}} + {\text{ H}}_{{2}} {\text{O }} \to {\text{ 3HNO}}_{{2}}$$

$${\text{HNO}}_{{2}} + {\text{ H}}_{{2}} {\text{O}}_{{2}} \to {\text{ HNO}}_{{3}} + {\text{ H}}_{{2}} {\text{O}}$$

The main enzymatic generators of H_2_O_2_ in the human body are nicotinamide adenine dinucleotide phosphate oxidases (NADPH oxidases) and mitochondrial respiratory chains, as well as various other oxidases. NADPH oxidases produce a superoxide anion (O_2_^−^), which can be converted to H_2_O_2_ by superoxide dismutase or spontaneously [10]. The important quantitative reduction system of methemoglobin requires nicotinamide adenine dinucleotide (NADH), which is produced by the Embden–Meyerhof glycolytic pathway and NADPH, which is produced by the pentose phosphate pathway [1, 11]. Activation of these pathways may be involved in the present patient’s results.

Also, the GO enrichment analysis showed that neutrophil-related pathways such as “neutrophil degranulation” and “neutrophil-mediated immunity” were activated. Mumby *et al*. reported that methemoglobin activates the NF-κB and MAPK pathways in human lung epithelial cells, leading to the release of IL-8 and MCP-1 [12]. This might support our finding that neutrophils were activated in the pathogenesis of our patient’s methemoglobinemia.

The strength of this case report lies in its potential to unveil a cytopathologic mechanism of methemoglobinemia through RNA-seq. Nonetheless, a significant drawback of this report is that the results of RNA sequencing may not have direct clinical application in patients with methemoglobinemia. Moreover, the limited scope of this report focusing only on one case highlights the need for more extensive data collection to draw meaningful conclusions regarding the broader implications of this work.

## Conclusion

In this report, we evaluated for the first time RNA from the whole blood of a patient with methemoglobinemia. The RNA-seq analysis in the present case showed that an activated “hydrogen peroxide catabolic process” may be related to the pathogenesis of methemoglobinemia.

## Supplementary Information

**Additional file 1.** RNA Sequencing Methods.

## Data Availability

The raw data concerning this study were submitted under Gene Expression Omnibus accession number GSE200333 for future access.

## Abbreviations

CT: Computed tomography
GO: Gene ontology
NADH: Nicotinamide adenine dinucleotide
NADPH oxidases: Nicotinamide adenine dinucleotide phosphate oxidases
RNA: Ribonucleic acid
SpO_2_: Oxygen saturation
