# Supplementary material for: Whole-blood ribonucleic acid sequencing analysis in methemoglobinemia: a case report
Source: J Med Case Rep. 2023 Jun 10;17:238. doi: 10.1186/s13256-023-03976-0 (PMC10257280; doi:10.1186/s13256-023-03976-0)
Supplement: Supplementary file 1 — Additional file 1. RNA Sequencing Methods. [file 13256_2023_3976_MOESM1_ESM.docx]

*RNA Sequencing Methods*

Total RNA was isolated from the whole blood of the patient using a PAXgene™ Blood RNA System (BD Biosciences, Franklin Lakes, NJ, USA). The collection tubes containing the blood samples were stored at -30°C until the analysis. The eluted RNA was dissolved in RNase-free water. The quality and quantity of RNA were evaluated using a Bioanalyzer 2100 system (Agilent Technologies, Santa Clara, CA, USA). The RNA was converted into double-stranded cDNA libraries using a SMART-seq HT kit (Takara, Shiga, Japan) according to the manufacturer’s protocol. The libraries were quantified using the Illumina Library Quantification Kit (Kapa Biosystems, Wilmington, MA, USA), and the fragment size distribution was determined using a bioanalyzer.

High-throughput sequencing was performed using a MGIseq 2000 system (MGI Tech Co., Ltd., Shenzhen, China) with 100-bp paired-end reads, which were converted into fastq files. Tophat2 [1] was used for read alignments using the human reference genome (hg19). BAM files were converted to raw count files using featureCount [2]. The raw counts were analyzed using iDEGES/edgeR in the TCC package [3].

From the RNA-seq results, only RNAs with protein coding were extracted based on the Ensembl database [4]. The top 2000 RNAs with the highest mean absolute deviation (MAD) and a |fold change| of ≥1.5 between day 5 and day 1 were selected for the analysis. A Gene Ontology (GO) enrichment analysis of the analyzed RNAs was performed. The GO enrichment analysis was conducted using the R-package clusterProfiler [5].

**References**

1. Kim D, Pertea G, Trapnell C, Pimentel H, Kelley R, Salzberg SL. TopHat2: accurate alignment of transcriptomes in the presence of insertions, deletions and gene fusions. Genome Biol. 2013;14(4):R36. https://doi.org/10.1186/gb-2013-14-4-r36.

2. Liao Y, Smyth GK, Shi W. featureCounts: an efficient general purpose program for assigning sequence reads to genomic features. Bioinformatics. 2014;30(7):923–30. https://doi.org/10.1093/bioinformatics/btt656.

3. Sun J, Nishiyama T, Shimizu K, Kadota K. TCC: an R package for comparing tag count data with robust normalization strategies. BMC Bioinformatics. 2013;14:219. https://doi.org/10.1186/1471-2105-14-219.

4. Ensembl genome browser 105. https://www.ensembl.org/index.html?redirect=no. Accessed 18 Mar 2022.

5. Yu G, Wang LG, Han Y, He QY, clusterProfiler: an R package for comparing biological themes among gene clusters. OMICS. 2012;16(5):284–7. https://doi.org/10.1089/omi.2011.0118.
